# What Neuroscientific Studies Tell Us about Inhibition of Return

**DOI:** 10.3390/vision3040058

**Published:** 2019-10-29

**Authors:** Jason Satel, Nicholas R. Wilson, Raymond M. Klein

**Affiliations:** 1 Division of Psychology, School of Medicine, College of Health and Medicine, University of Tasmania, Launceston, Tasmania 7250, Australia; nr.wilson@utas.edu.au; 2 Department of Psychology and Neuroscience, Faculty of Science, Dalhousie University, Halifax, NS B3H 4R2, Canada; ray.klein@dal.ca

**Keywords:** inhibition of return, oculomotor system, orienting

## Abstract

An inhibitory aftermath of orienting, inhibition of return (IOR), has intrigued scholars since its discovery about 40 years ago. Since then, the phenomenon has been subjected to a wide range of neuroscientific methods and the results of these are reviewed in this paper. These include direct manipulations of brain structures (which occur naturally in brain damage and disease or experimentally as in TMS and lesion studies) and measurements of brain activity (in humans using EEG and fMRI and in animals using single unit recording). A variety of less direct methods (e.g., computational modeling, developmental studies, etc.) have also been used. The findings from this wide range of methods support the critical role of subcortical and cortical oculomotor pathways in the generation and nature of IOR.

## 1. Introduction

An inhibitory aftermath of orienting was discovered in the early 1980s by Posner and Cohen [1] and subsequently named “inhibition of return” (IOR) by Posner et al. [2]. In these early papers, Posner and colleagues proposed a novelty-seeking function for IOR and in the next decade or two the phenomenon was subject to intense investigation. Eventually, a variety of exciting behavioral findings provided converging evidence for this proposal. Beginning with Posner et al. [2], a wide range of neuroscientific tools have been utilized to explore the neural basis of IOR, and in some cases, to resolve questions that arose primarily from behavioral studies. Our goal in this paper is to describe what we have learned about IOR from this neuroscientific literature. To provide the context for our coverage of this neuroscientific literature, we will begin with a brief overview of IOR’s behavioral manifestations including its cause and effects, its spatial and temporal properties, its role as a foraging facilitator (see [3], for a review that despite its age remains quite contemporary), and of disputes in the IOR literature that neuroscientific evidence might help to resolve.

## 2. Behavioral Manifestations

In a review such as this one, it is prudent to begin by noting that there is no widespread agreement about the nature of IOR [4]. The source of some disagreements (as anticipated by Klein [3]) may be in overextension(s) of the term. Most notably, a cue-induced reaction time delay in the processing of a subsequent target can be due to sensory adaptation when it is presented along the same pathway traversed by the cue [5], particularly when the interval between cue and target onset is ~500 ms or less. When such an “inhibitory” effect (also called “onset detection cost”) is conflated with IOR there is bound to be confusion. Here, we will focus on the longer-lasting inhibitory aftereffect of orienting, which we believe comes in two forms (first clearly distinguished in [6]) depending on whether the reflexive oculomotor system is in a suppressed or activated state when it is generated. As described in their account of the history of IOR (Hilchey et al. [5]; see also [7]), this duality has engendered confusion since the phenomenon was named by Posner et al. [2].

### 2.1. Causes and Consequences

Possible causes of IOR include prior orienting of attention, peripheral stimulation, and activation of the oculomotor system. Posner and Cohen [1] rejected the attentional cause because they demonstrated that prior endogenous allocation of attention did not generate IOR, a finding that has been subsequently confirmed (e.g., Rafal et al. [8]). They endorsed sensory stimulation as the cause because they believed that IOR was generated when peripheral cues were balanced around fixation. However, with better control conditions, this finding was not supported [9] and Posner et al. [2] further demonstrated that IOR could be generated by an endogenously directed eye movement in the absence of a unique peripheral onset. Using visual, auditory, and tactile stimuli and a target-target design, Spence et al. [10] found that as long as a target was presented in the same spatial position as the previous one (regardless of whether the modality repeated or switched to a different modality), there was a reaction time delay compared to when the spatial location changed. This finding, that IOR occurs cross-modally between all pairings of vision, audition, and touch, suggests that IOR is not restricted to cues and targets being delivered along the same sensory pathway. Early studies [2,8] suggested that the cause of IOR was activation of the oculomotor system and even though endogenous preparation of an eye movement does not cause IOR [11], this is still the most likely cause.

Three broad loci have been proposed for the effects IOR might have on subsequent behavior at, or near the inhibited location—it could degrade or delay sensory processing, delay or discourage spatial responses, or delay or discourage the orienting of attention. Of course, these possible loci are not mutually exclusive and a direct inhibitory effect at one level of processing could result in indirect inhibitory effects at other levels. Particularly, whether IOR affects input or output levels of processing may depend on the activation state of the reflexive oculomotor system [12]. According to this view, when this system is suppressed, inputs or attention are affected; when it is not suppressed, IOR manifests as an output level response bias.

### 2.2. Spatio-Temporal Properties

The time course of IOR is usually measured in a paradigm wherein spatially uninformative peripheral cues precede the target by varying intervals. In this paradigm, as demonstrated in Samuel and Kat’s [13] review, IOR can last for at least three seconds. The apparent onset of IOR depends on several variables, including the complexity of the task, whether the target is accompanied by distractors, and the importance of shifting attention away from the cue. As noted by many authors: “It is possible that inhibition is present but will not be seen in performance because its effects are obscured by other processes (such as facilitation or a response-repetition advantage) operating at the same time as the hypothesized inhibition” [3] (p. 141). Therefore, one explanation for the variability in the apparent onset IOR depends on the time required for attention to disengage from the location of the spatially uninformative cue where facilitation due to attentional capture might obscure any inhibitory effects of the cue. Moreover, processes that have a negative effect on reaction time may masquerade as IOR, leading to incorrect inferences about how early IOR begins when these processes operate as an immediate consequence of the cue (e.g., sensory adaptation [5] and onset detection cost [14]).

In terms of the spatial extent of IOR, when generated by a stimulus at a particular location in the periphery (i.e., the cue), many studies have reported a gradient of decreasing inhibition centered on this location (e.g., [15]; for a recent review see [16]). Interestingly, when IOR is generated by an array of a small number of cues, the location that seems to be most inhibited is the geometric midpoint of the cues (likened to a “center of gravity”), even if no stimulus was presented there [9,17]. Using a 50 ms interval between such an array of cues and a target calling for a saccade, Christie et al. [18] discovered an inverse effect. At this very short cue-target interval, rather than being inhibited, eye movements were facilitated toward the center of gravity of the array. This finding provides evidence for the proposal that prior activation of the oculomotor system is the cause of the subsequent IOR.

As first demonstrated by Maylor and Hockey [19], when the eyes move between the presentation of a cue and a target, the target suffers from greater inhibition at the spatiotopic location of the cue than at its retinotopic location. More recently, it has been demonstrated by Pertzov et al. [20] and Hilchey et al. [21] that spatiotopic coding was present as early after the saccade as they tested, and Yan et al. [22] demonstrated that spatiotopic recoding happens just before the eye movement. Furthermore, when a cued object moves between the cue and target, IOR can be seen at the new location of the cued object rather than, or in addition to the originally cued location [23]. From these findings, it has been inferred that IOR is coded in a scene- or object-based representation. Supporting this inference are the findings in both visual search (for a review see [24]) and cue-target paradigms [25] that IOR is eliminated if the scene is removed when the target is presented.

### 2.3. IOR as Foraging Facilitator

In Posner’s seminal studies [1,2], the function of IOR was proposed to be novelty seeking, and in [2] such novelty seeking was hypothesized to increase the efficiency of visual search by encouraging orienting toward new items. This proposal was supported by later studies that looked directly for IOR within visual search paradigms. Using a probe-following-search task, Klein [26] found inhibition at the locations attention had presumably visited during a search to determine that the items (distractors) were not the target. Klein and MacInnes [27] replicated this finding in a more ecologically valid, “camouflaged” search paradigm, employing scenes from “Where’s Waldo”. One major finding of such studies is that inhibition can be observed at multiple previously fixated locations. Based on these findings, Klein elaborated on Posner and Cohen’s [1] proposal, theorizing that IOR acts as an inhibitory tagging system that marks multiple previously attended locations and proposed that such a system could function as a foraging facilitator [26,27]. For a review of IOR findings in visual search tasks, see [24].

## 3. Neuroscience

Due to the temporal characteristics of IOR, neuroscientific research has been largely reliant on the high temporal resolution of ERP techniques to investigate the neural substrate of IOR. However, other methods have also been employed, including studies with patients who have subcortical brain injuries, developmental studies on infants, fMRI, TMS, single-cell recordings of rhesus monkeys, and computational modelling. Here, we review research in these areas.

### 3.1. Patient Studies

Early findings [2] from patients with subcortical damage due to progressive supranuclear palsy and cortical lesions involving the parietal or frontal lobes suggested that subcortical, but not cortical, systems were involved in the manifestation of IOR. The involvement of one subcortical structure that is at the nexus of control of the oculomotor system, the superior colliculus (SC) was later confirmed to be critical for the generation of IOR in patients with localized damage to this structure [28,29]. As described next, cortical involvement was later demonstrated to also be important for the coding of IOR in environmental and object (or scene) coordinates. In retrospect, perhaps this is not surprising, once IOR had been found to be represented in these coordinates, because the SC controls eye movements in oculocentric (or retinotopic) coordinates.

Tipper and colleagues [30] employed the moving-objects paradigm in two split brain patients to explore cortical contributions to object-based IOR. They demonstrated that an intact corpus callosum is necessary to transfer object-based inhibitory tags from one hemisphere to the other. In a study of patients with visual form agnosia, Smith et al. [31] demonstrated that although object-based facilitation effects were impaired, object-based IOR remained intact.

Sapir et al. [32] employed a version of the Maylor and Hockey [20] paradigm, in which a saccade intervenes between the cue and target to explore environmental coding of IOR in patients with lesions to the right intraparietal sulcus. A cue was presented above or below fixation and participants either remained fixated or made a leftward or rightward saccade in response to a central arrow. Normal participants and patients showed similar magnitudes of IOR in the stationary condition. When eye movements were made, the target could appear at either the retinal or environmental location of the cue. Normal participants showed IOR at both locations, but patients with right parietal damage only exhibited inhibition at the retinal location of the cue. This finding suggests that circuitry in the parietal lobes is responsible for preserving, in environmental coordinates, the inhibitory tags laid down by the SC.

An interesting response modality dissociation was reported by Bourgeois et al. [33]. Patients with damage to the right hemisphere were tested using a target-target paradigm in which IOR was expected when the current target was presented in the same location as the previous target. One condition required manual responses when a target was detected; the other condition required saccadic responses to targets. In a group suffering from left neglect associated with right parietal damage and/or disconnection of parietal from frontal regions, IOR was observed in the good (right) visual field when saccadic responses were required. In striking contrast, when manual responses were made, instead of suffering from IOR, repeated targets in the good visual field benefited from facilitation. It was suggested that with manual responses, IOR depends on an intact cortical circuit (fronto-parietal attentional network) in the right hemisphere whereas with saccadic responses, IOR might be mediated by subcortical circuits (retinotectal visual pathway).

Smith et al. [34] tested a neurologically normal individual (AI) who was unable to make eye movements due to congenital opthalmoplegia (oculo-muscular atrophy). Whereas AI could orient her attention endogenously in response to informative central cues, her attention was not captured exogenously by uninformative peripheral cues. Nevertheless, such cues did generate significant IOR. Converging evidence for this dissociation (IOR without exogenous cueing) was obtained by Smith et al. [35] using eye abduction in normal participants. With this manipulation, wherein cues and targets can be presented at locations that the eyes cannot reach, normal IOR and endogenous orienting were observed, but attention was not exogenously captured by peripheral cues. Although we are aware of one study with conflicting results [36], the discrepancy may be rooted in the difficulty of firm conclusions from the eye-abduction manipulation [37].

### 3.2. Developmental Studies

**Figure 1:**
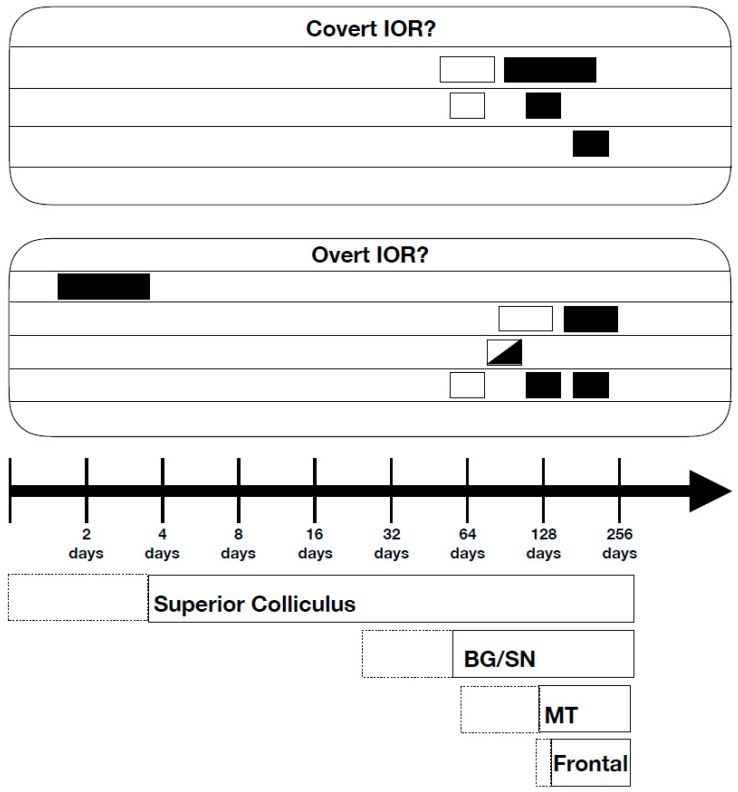
The relative time-course of maturation of different systems involved in orienting, as discussed by Johnson [39], is shown by the rectangles below the timeline (dotted lines simply reflect inter-individual variability in system maturation). These include the superior colliculus, the basal ganglia (BG), the substantia nigra (SN), the MT, and the frontal lobes. All the experiments illustrated here measured IOR using eye movements. Studies finding IOR (black rectangles) and failing to find IOR (open rectangles) in infants of different ages with covert (no eye movement to the cue) and overt (eye movements to the cue) orienting as the causal event are shown above the timeline. The split rectangle of reflects the fact that IOR was obtained when target eccentricities were 10 deg, but not when they were 30 deg. This figure has been redrawn from [38].

Although most research on IOR in normal individuals has been conducted with college age participants, there are data on the early development of IOR and on how it might change with aging. Research on the early development of IOR, as summarized by Klein [38] is illustrated in Figure 1, along with Mark Johnson’s [39] analysis of the relative rates of development of neural circuitry that controls orienting in adults. Among the important developmental findings is the observation of IOR in newborns less than four days old [40,41]. Because subcortical pathways are operational in infants whereas the cortex is still developing, this finding supports the conclusion from patient and behavioral studies that the SC is critical for the generation of IOR. It is noteworthy that in these newborn studies the effect of IOR was generated and measured with oculomotor behavior. Thus, in the context of the 2-forms of IOR described above, this form, almost certainly, would have been the output form.

Surprisingly, the IOR that is seen in newborns is absent in 1–2 month olds. According to Johnson et al. [42], inhibitory projections through the basal ganglia and substantia nigra that regulate SC activity become functional at around one month of age. It is thought that the development of this pathway, which encourages obligatory attention and response repetition would work against IOR. The subsequent development of frontal systems that control the inhibitory projections to the SC from the basal ganglia/substantia nigra is thought to mediate the reappearance of IOR at around 3–4 months of age. In later childhood, adulthood, and in studies of aging, IOR may not be seen or its appearance may be delayed when, for a variety of reasons, after capture of attention by the spatially uninformative peripheral cue, attention lingers or fails to disengage from this location [38].

### 3.3. Human Brain Imaging

IOR has been extensively investigated with human brain imaging, particularly with electro-encephalography (EEG) using the event-related potential (ERP) technique. The vast majority of these studies have used a traditional Posner cueing task with peripheral stimuli requiring manual responses to the targets. When this literature was reviewed by Martin-Arevalo et al. [43], it was concluded that there was not one single ERP component that could serve as a “marker” for IOR. Although reduction of the early sensory P1 component was often seen in the literature reviewed by Martin-Arevalo et al. [43], P1 reductions are an unlikely reflection of IOR because, as was pointed out by Satel and colleagues [44,45], these modulations can occur without IOR and IOR can occur without P1 reductions.

Martin-Arevalo et al. [43] excluded a few studies from their review that required participants to make eye movements at some point during a trial. Indeed, in all included studies, participants were instructed not to make eye movements and researchers typically removed the data from any trials with obvious eye movements. One reason that there have been so few EEG studies investigating IOR with activated oculomotor systems is that eye movements can contaminate the ERPs due to the low signal-to-noise ratio of this technique [46]. However, as noted above, suppressing the reflexive oculomotor system in this way might lead to IOR affecting the input stages of processing rather than the output stages. These studies, which involved suppressed reflexive oculomotor systems have often found a reduction in the amplitude of the P1 ERP component for cued relative to uncued peripheral targets. Furthermore, this P1 effect has been shown to be correlated with behavioral measures of IOR [43,44].

However, there have also been a few ERP studies that have allowed eye movements in response to the cues, activating the oculomotor system on each trial. When cues and targets were both peripheral stimuli and eye movements were made to cues [44], although P1 effects were still observed, the P1 modulation was not correlated with behavioral IOR, unlike the case when the eye movement system was suppressed in this and in the earlier studies. Furthermore, this P1 cueing effect disappeared entirely when there was no repeated peripheral stimulation (i.e., eye movements were made in response to central arrow cues) [45], even though IOR was still observed behaviorally in response to the peripheral targets. Similarly, when the spatiotopic location was dissociated from the retinotopic location with an eye movement between cue and target, greater behavioral inhibition was observed at the spatiotopic location than at the retinotopic location, however cue-related P1 reductions were only observed in the retinotopic and not in the spatiotopic condition [46].

A later ERP component in the 220–300 ms post-cue period, the Nd, is also often modulated by cueing in IOR paradigms [43]. In addition to being observed when eye movements are forbidden and there is repeated peripheral stimulation, Nd modulations have also been observed when eye movements were made in response to central arrow cues [45] and at spatiotopic, but not retinotopic locations when an eye movement occurred between cue and target appearance [46]. However, these Nd effects are even more inconsistent than those related to P1. They are not always present and sometimes go in the ‘wrong’ direction, suggesting that although something is going on in this time range, the Nd component may not be the most appropriate marker for IOR [45].

Other studies, e.g., [47,48] have explored the possibility of IOR modulating the amplitude or latency of the N2pc component, which arises in a similar time range as the Nd component and is assumed to reflect a shift of attention. In the first such study, McDonald et al. [47] discovered that the N2pc component was reduced, but not delayed for targets presented at the cued location. Using a visual search paradigm, Pierce et al. [48] obtained converging evidence for this finding. As yet, we are not aware of any studies with eye movements that have investigated the association of N2pc modulations with IOR.

The majority of these ERP studies have focused on the brain’s response to targets that might or might not have been suffering from IOR. It is important to point out that, in the cue-target paradigm, IOR is generated by the cue and measured by the target. The emphasis on the target in ERP experiments is designed to elucidate the nature of IOR’s effect(s) on processing. Fewer studies have focused on the brain’s response to the cues, an emphasis that, in principle, can tell us about the nature of IOR’s cause(s). Using such an alternative approach to investigate neural activity during the cue-target period, Tian et al. [49] developed a theoretical model of IOR. This work attempted to identify the areas activated at different stages after cue onset using LORETA (low-resolution brain electromagnetic tomography) source localization algorithms. The main idea is that attending to cued stimuli stimulates neurons in early visual areas including the SC, which then sends signals to cortical areas such as the parietal and frontal eye fields, generating inhibitory tags that represent previously attended locations. These tags then feed back down to the SC, inhibiting subsequent eye movements to the inhibited locations (i.e., IOR).

Another alternative approach to investigating sensory activity during the cue-target interval is to use the steady-state visual evoked potential (SSVEP) technique [50]. SSVEPs are periodic electrophysiological signals in the input pathway that are evoked by periodic stimulation and that share the same frequency as the stimulus. For visual stimuli, SSVEPs are observed over occipital cortex. A number of studies have demonstrated that these SSVEP signals are modulated by spatial attention (i.e., the signal is enhanced when attended), e.g., [51,52]. As far as we know, only one study [53] has employed this technique to explore the sensory consequences of an uninformative peripheral cue. This study found a biphasic pattern with enhanced signals from the cued location immediately after the appearance of the cue reversing to suppressed signals beginning about 200 ms later. Although the latter finding was assumed to be a reflection of IOR, this is challenged by the fact that IOR was observed behaviorally (in simple RT to targets) at 1200 ms post cue onset while the SSVEP suppression at the cued location was no longer present after 800 ms. We believe, therefore, that the SSVEP suppression observed at the cued location might have been a reflection of sensory adaptation. Regardless of one’s interpretation, further research using this powerful methodology would be welcome.

In addition to using EEG, a few studies have also used the fMRI technique in an attempt to identify the neural circuits associated with IOR. Due to its low temporal resolution, fMRI is a poor method for exploring IOR. Moreover, as noted by Klein (2004, pp. 552 [54]): “it is difficult to generate a reasonable pair of conditions to generate a subtraction that might tap into the presence of IOR. This is because IOR may be generated by a cue whether or not a target is presented and regardless where it is presented and IOR is just as likely to follow orienting to a target as to follow orienting a cue [6].” Müller and Kleinschmidt [55], however, conducted a clever fMRI experiment, aimed at determining whether IOR might have a negative impact on processing in early visual pathways. They avoided sensory cue-target interactions on cued trials by presenting the target close to the cue but on the opposite side of the vertical meridian (i.e., opposite hemifield) and they compared the activity in occipital cortex for cued versus uncued targets. Strongly supporting an input form of IOR, they found that the responses in occipital areas stimulated by the target were reduced for targets suffering from IOR. In this study, although participants were not given trial-by-trial feedback on their oculomotor behavior in the magnet, they were trained with eye monitoring before the fMRI session and according to the authors, made eye movements on less than 1% of the trials during training. Therefore, it seems likely that their reflexive oculomotor systems were relatively suppressed during the fMRI recording sessions. According to Klein and Redden’s view of the two forms of IOR [12], if an experiment like this were repeated with the IOR caused by a pro-saccade to the cue, suppression of visual cortex activity at the originally cued location would not be observed.

### 3.4. Manipulations Aimed at Exploring the Roles of Neural Structures and Pathways

Converging evidence for the special role of the superior colliculus in the generation of IOR can be found in two methods that have been used to differentially access this structure: nasal/temporal asymmetries and S-cone stimuli (for a review of the use of these methods in the study of visual attention, see [56]).

The retinotectal pathway sends visual inputs via the optic chiasm directly to the superior colliculus with more copious connections from the temporal hemifields (nasal hemiretinae) (for a review see [57]). Rafal et al. [8] hypothesized that if the SC played a special role in the generation of IOR, then stimulating a pathway with more copious connections to the SC ought to result in greater IOR. When they did this (Experiment 1, [8]) by presenting cues and targets monocularly they found, in accordance with their hypothesis, substantially more IOR when cues and targets were presented to the temporal hemifields than when they were presented to the nasal hemifields. Given our emphasis on the importance of the state of the reflexive oculomotor system for whether the input or output form of IOR might be generated, it is important to note that in this experiment eye position was not monitored and so it is likely that the output form was operating even though the response modality was manual.

An alternative strategy is to use S-cone stimuli that bypass the retinotectal pathway. Although such stimuli will not reach the SC directly, they will eventually get there via the geniculo-cortical pathway. Sumner et al. [58] used this strategy by presenting cues that were either luminant or S-cone followed 400–500 ms later by luminant targets. In one condition participants detected these targets using manual responses, and in another condition, they made saccades to the targets. With the typical luminant cues they found IOR regardless of the response modality. However, with S-cone cues, they only observed IOR when the eyes remained fixated for the duration of the task and target detection was signaled by a manual response. In other words, saccades were insensitive to the prior location of S-cone cues. This pattern of results suggests that the retinotectal pathway plays a special role in generating the output form of IOR when prosaccades are required. Moreover, if it is assumed that the reflexive oculomotor system was suppressed in their manual response condition, it would be reasonable to suggest that the input form of IOR was generated in the manual response condition.

Transcranial magnetic stimulation (TMS) is a powerful tool for inferring whether a targeted brain structure plays an important role in a particular behavior. A few studies have employed TMS in an effort to deduce which structures in the brain are integral to IOR. Three studies [59,60,61] administered TMS pulses over various brain structures on each trial during the time between the presentation of the cue and the target. Thus, if a certain structure was integral in generating IOR (through exposure to a cue), the TMS pulse to that area should nullify its effect and IOR would not be observed. Indeed, TMS pulses to the right frontal eye field [59], the temporoparietal junction (TPJ) [60], and the right intraparietal sulcus (IPS) [60] interfered with IOR. Importantly, using TMS in conjunction with a retinotopic/spatiotopic reference framing spatial cueing paradigm (as described in Section 2.2), van Koningsbruggen et al. [61] found an asymmetrical functionality for the right anterior intraparietal cortex. They showed that TMS pulses to this region diminished spatiotopic inhibition for targets both ipsilateral and contralateral to the pulse. Conversely, pulses to the left anterior intraparietal cortex had no effect on the usual IOR pattern. This finding provides strong converging evidence for the conclusion from patient work [33] described earlier, that the right parietal cortex is the neural substrate responsible for updating the locus of IOR to a spatiotopic representation in the presence of eye movements.

Seeking support for the response-modality dissociation reported in Section 2.2, Bourgeois et al. [62] administered 1200 TMS pulses over a 20-min period to create a temporary and reversible lesion of the right IPS or right TPJ. Similar to the findings from patients with neglect, for right-sided targets TMS-mediated disruption of either area decreased or eliminated manual, but not saccadic, IOR. By contrast, a later study by Bourgeois et al. [63], that used TMS to disrupt the left TPJ or left IPS showed no change in the IOR pattern for either manual or saccadic responses. Taken together, these results suggest both an asymmetrical control of visuospatial attention by the right parietal cortex and add converging evidence for the view that IOR may depend on different neural circuits depending on the activation state of the reflexive oculomotor system.

In an ingenious experiment, Gabay et al. [64] exposed the archer fish (which gets its name from the fact that when foraging for food it shoots down prey on low hanging vegetation by spitting water) to a Posner cueing paradigm. Fish are an interesting species for drawing inferences about neural structures required for generating IOR because they have such an underdeveloped cortex. Cues and targets were presented on a monitor mounted over the tank in which the fish were swimming and the latency of accurate spitting was measured. When the fish successfully shot a stream of water at the target on the screen, some food was dropped into the tank. Demonstrating IOR, when the interval between the cue and target was greater than one second, the spitting was slower for targets at the cued than at the uncued location. This finding supports the observation of IOR in newborn infants (see section above demonstrating that the generation of IOR does not require a fully developed cortex). In a subsequent study [65], archer fish were exposed to an endogenous version of the Posner cueing task in which the color of a central stimulus indicated the likely side of the upcoming target. The archer fish showed early facilitation, which the authors attributed to learning rather than volitional control. Interestingly, IOR was observed at later intervals. The authors concluded that when orienting is generated subcortically (as would be the case in this primarily sub-cortical species), IOR is observed even if the cue had been presented centrally.

### 3.5. Monkey Neurophysiology

A number of monkey single cell recording studies have recorded from oculomotor areas such as the superior colliculus (SC), while the animals performed spatial cueing tasks. Dorris et al. [66] demonstrated that at a CTOA of 200 ms, behavior was inhibited on cued trials as compared to uncued trials (as with humans), and that the activity of neurons in the SC was attenuated at the cued location (i.e., the target-related activity of neurons was lower when they had been previously been stimulated by a cue at the same location). Furthermore, when the same neurons were stimulated electrically (through the recording electrode), rather than by a visual stimulus to induce a saccade, facilitation rather than inhibition was observed, suggesting that the SC was not directly inhibited [65]. In later work [67], inhibition was observed behaviorally in monkeys at later CTOAs (100, 200, 500, and 1200 ms) while recordings were collected from both visual and visuomotor neurons in the SC. As in the previous study, target-related activity was reduced for cued neurons at 100 and 200 ms CTOAs, however, at the longer CTOAs this input attenuation was eliminated. These results suggest that the reduced responses of previously cued neurons in the SC at relatively short CTOAs are a reflection of sensory adaptation in the pathway projecting to the SC, whereas behavioral IOR observed at longer CTOAs reflects delays in pathways outside the SC.

Further neurophysiological data derived from recordings in monkeys provide additional converging evidence that, although the SC is crucial to the generation of IOR, higher cortical areas contribute importantly to output-based, oculomotor IOR. Mirpour and Bisley [68] recorded in the lateral intraparietal cortex (LIP) of monkeys while they performed a visual foraging task that allowed measurement of neural responses when new, or previously visited distractors entered the neuron’s receptive field. Providing a neural correlate for the suggestion that IOR might function as a foraging facilitator [2,26,27], it was found that responses were reduced for previously fixated as compared to new distractors. More recently, when recording from the FEFs during such a search task, Mirpour et al. [69] identified neurons that maintain increased activity throughout trials once the location they represent had been fixated. The authors proposed that these neurons keep track of all fixated stimuli, later sending these signals to priority maps in parietal cortex. Such priority maps in parietal cortex, driven by FEF signals, are a likely locus for the inhibitory tags leading to the output form of IOR.

### 3.6. Computational Modeling

As noted by Klein [54]: “What is most needed to advance our cognitive-neuroscientific understanding are some comprehensive and computationally explicit theories of the inhibitory aftermath of orienting” (p. 556). In real-world applications of visual search, such as robot navigation, inhibitory algorithms must be implemented in order to avoid perseverance on highly salient stimuli. However, such computations are normally implemented by simply reducing the salience of previously attended stimuli to zero for a few seconds [70], which is clearly not how the primate brain accomplishes the task (see [71] for a recent review of such salience models).

Neurobiologically plausible computational implementations of IOR have tended to use dynamic neural field models simulating the activity of neurons in the SC, based on data obtained from monkey neurophysiology and human behavior [72]. This work has shown great success in reproducing behavioral data in humans as well as monkeys and has played an important role in making predictions for further empirical work. Early simulations attempted to determine the extent to which sensory adaptation and emergent properties of saccade dynamics could account for the behavioral effects of IOR [73,74]. Although a great deal of data could be reproduced with such implementations, it was determined that IOR at CTOAs greater than around 1000 ms could not be explained or accurately reproduced with such input-based mechanisms. More recent implementations of this model have incorporated a later inhibitory mechanism (i.e., IOR), presumably via pathways from cortical areas such as the frontal eye fields and/or posterior parietal cortex [75].

In a complementary approach, diffusion modelling considers the accumulation of evidence toward some decision threshold. Here, the delayed responses for targets suffering from IOR might be explained by a variety of model parameters, e.g., see [76,77] including sensory-level effects (e.g., slower rate of accumulation) or a later decision-level effect (higher evidentiary threshold). Although diffusion modeling can be done without consideration of the neural circuits that mediate the behavior of interest, such models can be fruitfully linked and applied to specific neural circuits [78].

## 4. Conclusions

The neuroscientific research described here points to the critical role of the oculomotor system in the generation of output-based IOR that facilitates novelty seeking. IOR arising when the reflexive oculomotor system is not suppressed, is probably generated by projections from the SC to cortical areas (FEF, PPC [or LIP]) but not implemented in the SC. It is represented in spatiotopic coordinates, seems to arise only after about 600 ms post-cue and is likely represented in cortical areas affecting spatial responses regardless of the output modality (manual or oculomotor). When the reflexive oculomotor system is actively suppressed, however, the input-based form of IOR is generated, affecting early sensory pathways in retinotopic coordinates rather than response outputs. Early sensory adaptation also occurs along input pathways but only affects behavior for up to around 600 ms post-cue, and only when there is repeated peripheral stimulation. Further studies of the inhibitory aftereffects of orienting should be careful to disentangle these multiple inhibitory cueing effects.
